# FXR agonism protects against liver injury in a rat model of intestinal failure-associated liver disease

**Published:** 2017-10-15

**Authors:** Kiran V.K. Koelfat, Ruben G.J. Visschers, Caroline M.J.M. Hodin, D. Rudi de Waart, Wim G. van Gemert, Jack P.M. Cleutjens, Marion J. Gijbels, Ronit Shiri-Sverdlov, Rajeshwar P. Mookerjee, Kaatje Lenaerts, Frank G. Schaap, Olde Damink Steven W.M.

**Affiliations:** ^1^ Department of Surgery, Maastricht University Medical Center, Maastricht University, NUTRIM School of Nutrition and Translational Research in Me-tabolism, Maastricht, the Netherlands; ^2^ Tytgat Institute for Liver and Intestinal Research, Academic Medical Center, Amsterdam, the Netherlands; ^3^ Department of Pathology, Maastricht University Medical Center, Maastricht, the Netherlands; ^4^ Department of Medical Biochemistry, Academic Medical Center, Amsterdam, the Netherlands; ^5^ Department of Molecular Genetics, Maastricht University, Maastricht, the Netherlands; ^6^ Institute for Liver and Digestive Health, University College London, London, United Kingdom; ^7^ Department of Visceral- and Transplantation Surgery, RWTH Aachen University, Germany

**Keywords:** intestinal failure, liver disease, enterohepatic cycle, bile salt signaling, FXR, enterocutaneous fistula

## Abstract

**Background:**

Intestinal failure-associated liver disease (IFALD) is a clinical challenge. The pathophysiol-ogy is multifactorial and remains poorly understood. Disturbed recirculation of bile salts, *e.g.* due to loss of bile via an enterocutaneous fistula, is considered a major contributing factor. We hypothesize that impaired signaling via the bile salt receptor FXR underlies the development of IFALD. The aim of this study was to investigate whether activation of FXR improves liver homeostasis during chronic loss of bile in rats.

**Methods:**

To study consequences of chronic loss of bile, rats underwent external biliary drainage (EBD) or sham surgery for seven days, and the prophylactic potential of the FXR agonist INT-747 was assessed.

**Results:**

EBD for 7 days resulted in liver test abnormalities and histological liver damage. Expression of the intestinal FXR target gene *Fgf15* was undetectable after EBD, and this was accompanied by an anticipated increase in hepatic *Cyp7a1* expression, indicating increased bile salt synthesis. Treatment with INT-747 improved serum biochemistry, reduced loss of bile fluid in drained rats and prevented development of drainage-associated histological liver injury.

**Conclusions:**

EBD results in extensive hepatobiliary injury and cholestasis. These data suggest that FXR activation might be a novel therapy in preventing liver dysfunction in patients with intestinal failure.

**Relevance for patients:**

This study demonstrates that chronic loss of bile causes liver injury in rats. Abro-gated recycling of bile salts impairing of enterohepatic bile salt/FXR signaling underlies these pathological changes, as administration of FXR agonist INT747 prevents biliary drainage-induced liver damage. Phar-macological activation of FXR might be a therapeutic strategy to treat disorders accompanied by a per-turbed enterohepatic circulation such as intestinal failure-associated liver disease.

## 1. Introduction

Intestinal failure-associated liver disease (IFALD) is a feared complication in 40 to 55% of adult patients with intes-tinal failure due to e.g. short bowel syndrome or enterocuta-neous fistula (ECF) [1]. The clinical spectrum of liver disease in the context of intestinal failure is varied with signs of cho-lestasis, hepatic steatosis, steatohepatitis and fibrosis [1]. While multiple factors contribute to IFALD development, in-cluding intestinal anatomy, septic episodes, nutritional defi-ciencies and parenteral nutrition, the exact pathophysiology of IFALD remains poorly understood [2].

It has been postulated that loss of enteric fluid from pancre-atobiliary and intestinal secretions, contributes to the devel-opment of IFALD [3-5]. Thus far, only a few studies addressed the functional consequences of such loss. Rinsema et al. showed that loss of succus intestinalis in patients with an ECF was associated with development of hepatic damage [5,6]. Reinfusion of intestinal (*viz*. fistula) fluid into the distal small intestine improved liver injury, despite continued parenteral nutrition [5,6]. In particular loss of bile salts, a quantitatively important constituent of enteric fluid, was suggested to con-tribute to the development of liver injury in patients with an ECF [5]. Reinfusion of intestinal fluid into the distal enteric tract of intestinal failure patients with a high-output double enterostomy, also led to (rapid) recovery of liver test abnor-malities [7]. Collectively, these data suggest that an intact en-tero-hepatic circulation is crucial to maintain liver homeostasis.

Bile salts act as endogenous activating ligands of nuclear and plasma membrane receptors expressed in numerous tissues, but in particular in the small intestine and the liver [8]. The farnesoid x receptor (FXR) is a bile salt-sensing transcription factor that plays a key role in the regulation of bile salt synthe-sis, lipid and carbohydrate metabolism, and is required for maintaining intestinal integrity and limiting toxic effects of bile salts [8,9]. Furthermore, activated FXR exerts anti-inf-lammatory actions by inhibition of NF-kB activity, a central player in inflammatory processes [10].

Previous studies established the role of the gut in regulating bile salt synthesis [11,12]. In the terminal ileum, FXR stimu-lates the production of the enteric hormone fibroblast growth factor 15 (Fgf15) and its human orthologue FGF19 [13,14]. This ileal-derived hormone represses the hepatic expression of the bile salt-synthetic enzyme, *Cyp7a1*. Studies in several an-imal models with an obstructed enterohepatic circulation showed that disruption of the FXR-Fgf15 axis was associated with development of (cholestatic) liver injury [15]. The effect of chronic loss of bile fluid on development of liver injury and the therapeutic effect of FXR activation in this setting, has not been addressed yet in an experimental model.

An abrogated entero-hepatic cycle is expected to result in impaired delivery of bile salt ligands to bile salt receptors, in particular FXR that are essential for intestinal and hepatic function. Thus, we hypothesize that loss of bile fluid leads to diminished activation of FXR, dysregulated bile salt homeostasis and compromised hepatic and intestinal integrity, events that could underlie the development of IFALD. The aim of the study was to investigate the effect of FXR agonism (INT-747, a.k.a. obeticholic acid/Ocaliva®.) on prevention of entero-he-patic dysfunction in a rat model of IFALD due to continuous loss of bile.

## 2. Methods

### 2.1 Animals and Experimental Procedures

Male Sprague Dawley rats (Charles River) weighing 300-350 grams, were housed under controlled environmental con-ditions in separate cages at the animal housing facility of Maastricht University. Animals had free access to regular chow and water throughout the experiment. The study was approved by the Animal Care Committee of Maastricht Uni-versity (DEC 2009-170).

After an acclimatization period of one week, external bili-ary drainage (EBD) was performed essentially as described by Kuipers et al. [16] In brief, rats were anesthetized with isoflu-rane, laparotomized, and the common bile duct was exposed and ligated at its distal part. A small incision was made in the duct at approximately 1 cm from the duodenum and a silicone drain (silclear tubing; ID 0.51mm, OD 0.94mm, Mednet GmbH, Germany) was inserted. The drain was attached to the bile duct and tunnelled subcutaneously from the abdomen to the skull. Subsequently, it was connected with a curved metal stent (using an adjusted 21 Gauge hypodermic needle) secured to the skull with fast curing acrylic powder (Simplex Rapid, Kemdent, UK). A second catheter (polyethylene; ID 0.76mm, OD 1.22mm, Smiths Medical, UK) connected the metal stent with a swivel (Instech Laboratories, NL) protected by a metal spring (Instech Laboratories, NL) [16]. The sham procedure followed the same procedure with manipulation of the com-mon bile duct but without ligation, incision and cannulation of the bile duct.

In a pilot experiment, rats underwent continuous EBD for three or seven days to investigate the severity of liver injury. Although inflammation was already apparent after three days, other signs of histological injury and abnormal biochemistry (cholestasis and hepatocellular damage) developed after 7 days of continuous EBD (data not shown). This duration was cho-sen for the intervention study. Thus, rats underwent EBD for 7 days or were sham-operated (n = 8 per group). Immediately after surgery, animals received a daily intraperitoneal dose of the FXR agonist INT-747 (10 mg/kg in vehicle, kindly pro-vided by Intercept Pharmaceuticals) or vehicle alone (corn oil with 5% DMSO). The final dose of INT-747 was administered 24 hrs before sacrifice. Bile production in the drainage groups was determined daily. Unimpeded bile flow was maintained throughout the experiment in all animals in the drainage groups.

All animals were weighed daily, and none of the animals experienced significant weight changes during the course of the experiment (data not shown). Two rats in the vehicletreated EBD group died as a result of biliary peritonitis, one rat in the agonist-treated EBD group died as a result of dehydra-tion, and two rats in the vehicle-sham group died because of abdominal wall dehiscence (n = 1) or unknown cause (n = 1).

At the end of the experiments, rats were anesthetized with isoflurane and sacrificed through aortic puncture between 8: 00 and 12: 00 AM. Blood was transferred to EDTA tubes and plasma was prepared by centrifugation. Terminal ileum and liver were harvested and portions were snap-frozen or pro-cessed for embedding in paraffin. Plasma and tissue specimens were stored at -80°C until analysis.

### 2.2 Biochemical Analyses

Liver damage was assessed by analysis of plasma levels of alanine aminotransferase (ALT), aspartate aminotransferase (AST), GGT, ALP, and total bilirubin (Synchron LX 20 system, Beckman Coulter, NL). Systemic inflammation was evaluated by measuring plasma IL-6 levels using ELISA (R&D Systems, Minneapolis, MN). Plasma levels of the enterocyte damage marker ILBP were determined by ELISA (Hycult Biotech, Uden, The Netherlands) [17]. Serum levels of the acute phase protein lipopolysaccharide-binding protein (LBP) were deter-mined by ELISA (Hycult Biotech, Uden, the Netherlands) [18]. Bile salts were extracted from liver as described previously [19]. Total bile salts in plasma and liver extracts were meas-ured by an enzymatic cycling method using the Total Bile Ac-ids Assay kit (Diazyme, San Diego, CA). Bile salt composition of liver extracts was determined as described previously [20, 21]. Serum 7ɑ-hydroxy-4-cholesten-3-one (C4, a surrogate marker of CYP7A1 activity) was measured by LC-MS after acetonitrile precipitation as described earlier [22].

### 2.3 Liver Histology and Morphometric Analysis

After deparaffinization and rehydration, H&E stained liver sections (4 μm thickness) were scored individually on a 0 to 4 scale for inflammation by a blinded pathologist (MJG). Score 0 indicating no inflammation; score 1 indicating mini-mal periportal inflammation; score 2 indicating mild inflam-mation (periportal); score 3 indicating moderate periportal and sinusoidal inflammation and score 4 indicating severe peri-portal and sinusoidal inflammation. Fibrosis was scored on Sirius Red stained sections with score 0 indicating no fibrosis; score 1 indicating mild periportal fibrosis; score 2 indicating moderate periportal fibrosis with minimal sprouting; score 3 indicating severe periportal fibrosis with moderate sprouting and score 4 indicating bridging fibrosis. Bile duct proliferation was examined by morphometric analysis of pan-cytokeratin stained (Dako) liver sections. In brief, cytokeratin-positive cell area (>5 μm2) and total cell area (H&E stained) were deter-mined in 10 random fields (Leica DM3000 microscope, 100x magnification) of each section by supervised analysis of auto-mated image processing (Leica QWin v3 software). Only tan-gentially cut bile ductules were analyzed.

### 2.4 Western Blotting

For immunoblot analysis, liver tissue was homogenized in lysis buffer (200 mM NaCl, 10 mM Tris, 5 mM EDTA, 10% glycerol, 1% NP-40, pH 7.5). 20 µg solubilized liver protein was separated by reducing SDS-PAGE and transferred to PVDF membrane. Following blocking of unoccupied binding sites with PBS containing 5% non-fat dry milk powder, mem-branes were probed with rabbit anti-rat Cyp7a1 (a kind gift of Dr H.M. Princen, TNO, Leiden, The Netherlands) and rabbit anti-mouse β-actin (Sigma) antibodies. Secondary detection consisted of horseradish peroxidase-labelled goat anti- rabbit IgG antibody (Jackson ImmunoResearch Laboratories, Inc.) and immunocomplexes were visualized using enhanced chem-iluminescence (Thermo Scientific). Three independent liver homogenates were analyzed per experimental group.

### 2.5 RNA isolation and Quantitative Polymerase Chain Reaction

Total RNA was extracted from liver or ileal tissue using TRI reagent (Sigma). 750 ng DNAse-treated RNA was con-verted to cDNA (iScript cDNA synthesis kit, Bio-Rad, Hercu-les, CA). qPCR reactions were conducted in a volume of 20 µl containing cDNA equivalent to 10 ng total RNA, 1x Absolute qPCR SYBR Green Fluorescein Mix (Westburg, The Nether-lands) and 150 nM of gene-specific primers (Eurogentec, The Netherlands) (Supplementary Table 1), and were performed in duplicate. Gene expression levels were determined with iQ5 software (Bio-Rad) using a ΔΔCt relative quantification model. The geometric mean of the expression levels of two reference genes (*Hprt* and *Rplp0*) was used as normalization factor, and values are graphically presented relative to median expression in sham-operated controls.

### 2.6 Intestinal permeability

Intestinal permeability was assessed by measuring release of horseradish peroxidase from everted segments of terminal ileum as described previously [17].

### 2.7 Statistical analysis

For histological analysis, multiple fields per section were scored and averaged per animal. Histological scores were tested for significance with the Fisher’s exact test. Effects of EBD or agonist treatment on serum biochemistry, mRNA ex-pression, morphometric parameters, intestinal permeability, enterocyte damage and systemic inflammation were evaluated with the Mann-Whitney U test for unpaired samples. A Bon-ferroni correction for multiple testing was applied where ap-propriate. Differences in daily bile production in the drainage groups were tested with repeated measures ANOVA. For visu-al purposes, data in graphs are presented as means +/- standard error of mean. *P*-values below 0.05 were considered statisti-cally significant. Statistical analyses were performed using GraphPad Prism 6.0 (GraphPad Software Inc., CA, USA) and SPSS 22.0 (IBM SPSS Inc, Chicago, Illinois, USA).

## 3. Results

### 3.1 Histological liver damage and cholestasis after continuous biliary drainage

**Figure 1.**
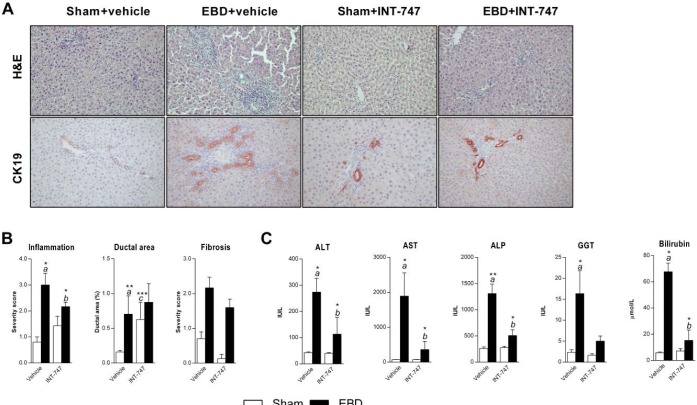
The effect of EBD on liver histology, liver tests and the effect of FXR agonism. Sham-operated rats (white bars) and rats undergoing external biliary drainage for 7 days (black bars, EBD) received vehicle or the FXR agonist INT-747 (n = 6-8 per group). (A) Representative histological images of H&E and CK19 stained liver sections. Note the portal inflammation, increased ductules and dilated cholangiocytes in drained animals receiving vehicle. (B) Histological scoring of inflammation and fibrosis, and morphometric analysis of ductal area. (C) Serum biochemistry of liver damage and cholestatic markers. *a* Indicates a significant effect of drainage in animals receiving vehicle. *b* Denotes a significant effect of INT-747 in drained animals. The signif-icance level is depicted by asterisks; *(P < 0.05), **(P < 0.01) and ***(P < 0.001).

**Figure 2.**
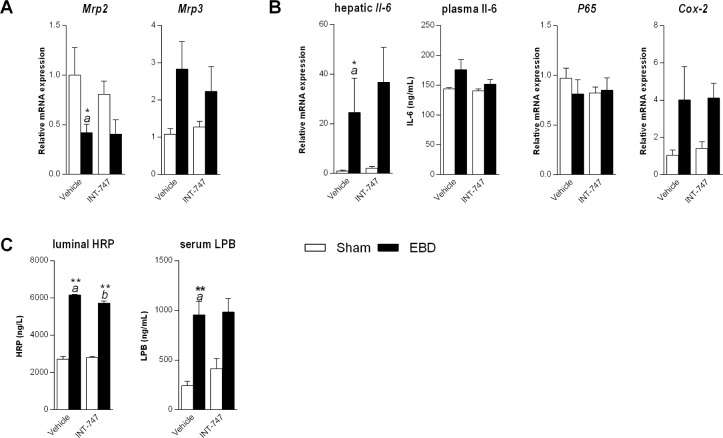
Effect of FXR agonism on liver histopathology induced by external biliary drainage. Sham-operated rats (white bars) and rats undergoing external biliary drainage for 7 days (black bars, EBD) received vehicle or the FXR agonist INT-747 (n = 6-8 per group). (A) Level of hepatic expression of Mrp2, Mrp3 and (B) NF-*k*B target genes and Il-6 in the circulation. (C) Intestinal permeability as assessed by horseradish peroxidase translocation in everted ileal segments and circulat-ing levels of the acute phase reactant LBP. *a* Indicates a significant effect of drainage in animals receiving vehicle. *b* Denotes a significant effect of INT-747 in drained animals. c Indicates a significant effect of INT-747 in sham-operated animals. The significance level is depicted by asterisks; *(*P* < 0.05) and **(*P* < 0.01).

Histological examination showed significant hepatic infla-mmation in the vehicle-treated EBD rats (Figure 1A). EBD was also associated with histological signs of biliary fibrosis (Figure 1B). Moreover, histological evidence for bile ductular proliferation was apparent, indicating injury to the biliary sys-tem (Figure 1A&B). Morphometric analysis revealed that the relative ductal area in liver sections was increased in EBD rats receiving vehicle (Figure 1B). Histological signs of inflamma-tion was accompanied by increased hepatic expression of IL-6 in drained animals receiving vehicle (*P* = 0.02, Fig 2B). A trend (*P* = 0.065) towards elevated circulating IL-6 was noted in drained animals receiving vehicle (Fig 2B). EBD resulted in cholestasis as judged from elevated plasma GGT, ALP and bilirubin levels, and suggesting altered hepatobiliary transport of cholephiles (Figure 1C). ALT and AST levels were significantly increased in the EBD -vehicle group reflecting hepato-cellular damage (Figure 1C).

### 3.2 Histological liver damage and cholestasis caused by con-tinuous EBD is ameliorated by FXR agonism

The consequences of activation of FXR on drainage-induced liver damage were studied by administration of the potent FXR agonist INT-747 [23] In contrast to biliary fibrosis, histo-pathological scores of hepatic inflammation were significantly lower in drained animals receiving INT-747 (Figure 1B). Morphometric analysis showed that the observation of an ex-panded ductular network after EBD was not counteracted by INT-747 administration in drained animals (Figure 1B). In fact, INT-747 treatment had a similar effect on ductular area in sham-operated animals (Figure 1B). Although treatment with INT-747 showed histological improvements, this was not ac-companied by a significant decrease in expression and circu-lating levels of IL-6 expression. Expression of other NF-κB target genes, i.e. the p65 NF-kB subunit and Cox2 was not affected by EBD or INT-747 treatment (Figure 2B).

Liver test abnormalities were largely (GGT) or even com-pletely (bilirubin, AST, ALT and AP) prevented by INT-747 (Figure 1C). To further investigate the drainage-associated cholestasis, we studied the expression of hepatic biliary trans-porters. The expression of multidrug resistance-associated protein-2 (Mrp2) was decreased after seven days of EBD indi-cating reduced capacity to secrete glucuronidated bilirubin into bile (Figure 2A). Among its numerous substrates, Mrp3 se-cretes bilirubin diglucuronide in the sinusoidal space. The ex-pression of the basolateral efflux pump *Mrp3* was unchanged after 7 days of EBD (Figure 2A). Despite clear effects of FXR agonism on abnormal liver tests, gene expressions of these transporters were not affected (Figure 2A).

### 3.3 Continuous biliary drainage is associated with increased intestinal permeability and is prevented by FXR agonism

Patients undergoing external biliary drainage develop bacte-rial overgrowth with bacterial translocation and endotoxemia, which can be prevented by reinfusing bile into the intestinal tract [24]. To further investigate the mechanism of liver injury in this model we explored the effect of chronic biliary drainage on intestinal permeability and presence of circulating LPB. Intestinal permeability, as assessed by translocation of horse-radish peroxidase in everted ileal segments, increased after 7 days of EBD (Figure 2C). This was accompanied by elevated serum LBP levels in drained animals (Figure 2C). This could likely initiate an inflammatory cascade which leads to hepato-cellular injury. INT-747 treatment did not affect intestinal permeability in sham-operated animals, but resulted in a re-duction of permeability (Figure 2C, *P* = 0.005) in drained an-imals without affecting circulating LBP (Figure 2C).

### 3.4 FXR agonism reduces biliary output in drained animals

**Figure 3.**
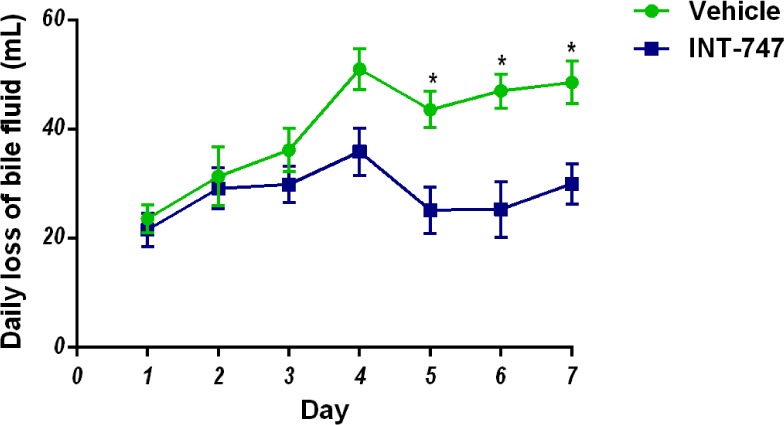
Effect of FXR agonism on biliary output. Daily production of bile during the course of external biliary drainage. The significance level is depicted by asterisks; *(P < 0.05).

Reinfusion of externally collected intestinal (viz. fistula) fluid back into the intestinal tract was shown to normalize the fistula output in patients with a high-output proximal ECF [6] To investigate the effect of FXR activation on the biliary out-put, externally drained bile was collected daily in drained ani-mals for 7 days. In INT-747 treated animals, drain output was reduced from day five onward (Figure 3). The latter suggests that biliary bile salt secretion, the main driving force for gen-eration of bile flow, is reduced following prolonged EBD in INT-747 treated animals.

### 3.5 Biliary drainage influences intestinal and hepatic FXR signaling and is largely restored by INT-747

**Figure 4.**
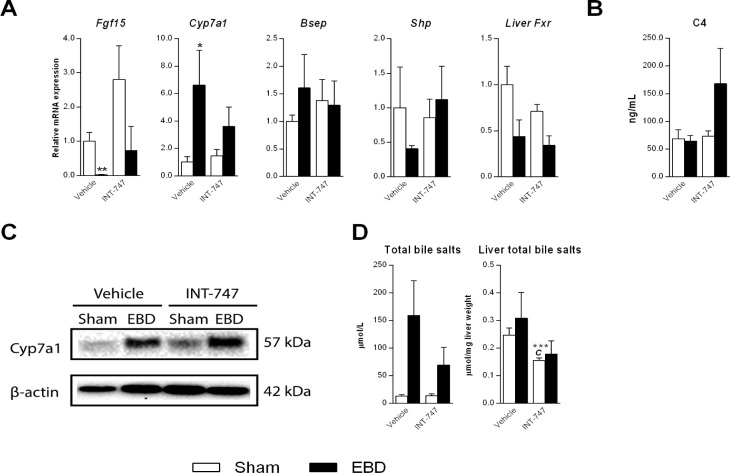
Effect of FXR agonism on drainage-induced FXR dysfunction. Sham-operated rats (white bars/symbols) and rats undergoing external biliary drainage for 7 days (black bars/symbols, EBD) received vehicle or the FXR agonist INT-747 (n = 6-8 per group). (**A**) Hepatic and ileal (only *Fgf15*) ex-pression of genes related to bile salt synthesis and transport. (**B**) Plasma C4 levels. (**C**) Representative immunoblot analysis of hepatic Cyp7a1 protein expression. (**D**) Total bile salt levels in the circulation and liver. a Indicates a significant effect of drainage in animals receiving vehicle. c Indicates a sig-nificant effect of INT-747 in sham-operated animals. The significance level is depicted by asterisks; *(*P* < 0.05), **(*P* < 0.01) and ***(*P* < 0.001).

FXR activation in this model was associated with improve-ment of liver test abnormalities, recovery from histological liver injury and reduced biliary fluid output. To study whether these beneficial effects could be attributed to restored bile salt homeostasis, we explored the FXR/Fgf15 axis in this model. Continuous EBD caused a pronounced decrease in intestinal mRNA expression of fibroblast growth factor 15 (Fgf15) and this was accompanied by increased *Cyp7a1* mRNA (Figure 4A) and protein (Figure 4C) expression, and apparent -yet statisti-cally not significant- elevation of serum bile salt levels in drained animals (Figure 4D), indicating impaired repression of this key bile salt synthetic enzyme by the regulatory intestinal FXR- Fgf15 axis. Despite increased transcripts of Cyp7a1 in drained animals, plasma C4 levels were similar in all groups (Figure4B). Hepatic bile salt levels were not affected by bili-ary drainage (Figure 4D). In sham-operated animals, INT-747 treatment resulted in reduced hepatic bile salt content (Figure 4D) . INT-747 treatment restored intestinal *Fgf15* expression and prevented the induction of *Cyp7a1* mRNA in drained ani-mals (Figure 4A). Nonetheless, Cyp7a1 protein levels re-mained elevated in drained animals receiving INT-747 treat-ment (Figure 4C). Despite the clear effects of INT-747 on se-rum biochemistry and histological scores in drained animals, and contrary to expectations, FXR agonism had no effect on expression of prototypical FXR target genes in the liver in-cluding *Bsep* and *Shp* (Figure 4A). A trend towards reduced hepatic FXR expression was observed in drained animals re-ceiving vehicle (*P* = 0.065), but FXR levels were similar in INT-747 treated groups of animals and their respective vehi-cle-treated controls (*P* = 0.44) (Figure 4A).

## 4. Discussion

Prolonged loss of bile fluid in patients with intestinal failure is associated with the development of liver disease in the con-text of intestinal failure [5]. The major finding of the present study is that hepatobiliary damage and cholestasis induced by prolonged external biliary diversion, can be prevented by treatment with an FXR agonist. These findings may be ex-plained by drainage-induced abrogation of FXR signaling re-sulting in deranged bile salt homeostasis. Cholestasis can give rise to bile salt-inflicted damage to the liver and/or biliary sys-tem. FXR agonism appears to re-instate normal feedback reg-ulation of bile salt synthesis via the intestinal FXR/Fgf15 axis.

Prolonged EBD resulted in damage to the liver (ALT and AST elevations) and the biliary compartment (ALP and GGT elevations). Hepatic inflammation may contribute to impaired canalicular transport (hyperbilirubinemia) and development and/or worsening of cholestatic liver injury [25-27]. Hepato-cellular and biliary damage triggers a reparative response (*i.e*. ductular reaction) that results in expansion of the biliary net-work [28, 29]. This is apparent after 7 days of EBD. FXR ago-nism, postulated to mimic restoration of bile salt signaling in drained animals, prevented most of the above histopathologi-cal alterations. Notably, INT-747 reduced inflammation in drained animals. INT-747 treatment also resulted in an en-larged ductular area. This may be due to the direct or indirect (via Fgf15) effects of activated FXR on cholangiocyte prolif-eration [30]. The apparent enlarged ductular area in sham-operated animals was not accompanied by alterations in inflammatory or fibrotic scores. This observation suggests that additional inflammatory triggers absent in sham-operated ani-mals are present (*e.g.* toxic bile salts, endotoxins and nutri-tional deficits like essential fatty acid deficiency) in drained animals. Indeed, hepatic *Il*-6 expression, a target of the NF-κB pathway [31], was elevated in drained animals but not detecta-ble in sham-operated animals. FXR is known to negatively regulate the NF-*k*B pathway, which is central to hepatic in-flammation [32]. Nonetheless, INT-747 did not lower hepatic expression of Il-6 or other NF-kB target genes. This may relate to the timing between last dosing of INT-747 and sacrifice, which may explain the general absence of clear transcriptional effects of FXR agonism. Alternatively, FXR agonism may reduce inflammation through NF-κB independent signaling pathways such as the c-Jun amino-terminal kinase ( JNK) sig-naling pathway [33]. Lack of obvious (long-lasting) transcrip-tional effects following INT747 administration (once daily as a bolus) appears to be a general pattern in this study, and may relate to the time interval of 12 hrs between last dosing and sacrifice. INT747 is administered in unconjugated form, and like other hydrophobic bile salts, is postulated to follow a nu-clear route after uptake by the liver [34]. By activating FXR, nuclear INT747 elicits a transcriptional response that aims to prevent bile salt toxicity, amongst others by promoting bile salt conjugation and accordingly aqueous solubility. INT747 is conjugated prior to secretion in bile and undergoes enterohe-patic circulation (predominantly in its conjugated form) in the sham- operated animals with intact biliary anatomy. In subse-quent rounds of hepatic transit, conjugated OCA is postulated to follow a non-nuclear route for rapid re-secretion in bile. Transcriptional responses elicited by INT747 in the liver may thus be of limited duration, i.e. only during first passage through the liver, in our experimental set-up. Functional con-sequences of transient FXR activation may persist for a longer period, as reflected in improved inflammatory scores and bili-ary fluid output (Figure 1B, 4) [34].

What could be the mechanism of EBD-induced liver dam-age? Failed delivery of activating ligands (viz. bile salts) re-sults in inadequate function of intestinal FXR during EBD. This potential mechanism has two functional consequences. Firstly, gut barrier integrity will become compromised as in-ferred from increased intestinal permeability [35], and sec-ondly, intestinal Fgf15-mediated regulation of bile salt synthe-sis will be disturbed. Impaired gut barrier function may give rise to translocation of bacteria and/or bacterial products re-sulting in portal endotoxemia and hepatic inflammation. This may be reflected by elevation of serum LBP, an acute phase reactant, and induction of hepatic *Il*-6 expression. Furthermore, inflammatory signaling in the liver may interfere with proper function of tight junctions between hepatocyte couplets or bile duct epithelial cells [36]. Similar to observations in other rat models, disrupted tight junctions can lead to bile regurgitation and inflammatory consequences [37-39]. Among other things, inflammation prevents the nuclear localization of Rxr-alpha [40], an obligate heterodimer partner for many nuclear recep-tors including FXR. This may underlie reduced hepatic Mrp2 expression after EBD, with retention of bilirubin evoking a compensatory secretion route via upregulation of *Mrp3*. Dis-turbed feedback regulation of bile salt synthesis on the other hand, results in enhanced production of bile salts as supported by elevated Cyp7a1 protein in drained animals. However, cir-culating C4 levels did not reflect the elevated Cyp7a1 protein. Although, INT-747 treatment had no significant effects on *Cyp7a1* mRNA/protein expression and levels of bile salts in the circulation and the liver, reduced bile flow suggests that FXR agonism prevents deregulated bile salt synthesis follow-ing prolonged EBD. Diminished bile salt synthesis in drained animals receiving INT-747 may result in reduced availability of substrates for Bsep and decreased biliary bile salt secretion. The latter constitutes the main driving force for bile formation.

The inflamed liver may be particularly sensitive to toxic ef-fects of excessive bile salts. Nonetheless, 7 days of biliary di-version did not affect hepatic bile salt content, nor did FXR agonism result in significant lowering of hepatic bile salts in drained animals. FXR controls the composition of the bile salt pool and regulates their conjugation, and accordingly influ-ences toxic potential of bile salt species [41, 42]. Drainage changed the composition of the hepatic bile salt pool towards a more hydrophilic, less toxic pool (data not shown). This is likely due to elevation of Cyp7a1 and increased synthesis of primary bile salt species, including tauro-β-muricholate. The hepatoprotective effect of INT-747 in drained animals appears unrelated to lowering of hepatic bile salt content or favorable changes in the composition of the hepatic pool (data not shown). The drainage-induced elevation of *Cyp7a1* is coun-teracted by INT-747, at least at the transcriptional level. Re-duced biliary output in drained animals receiving INT-747 can be interpreted as lowered bile salt synthesis and reduced availability to the canalicular transporters responsible for bili-ary secretion of these osmolytically active molecules. En-hanced bile salt synthesis in drained animals may result in det-rimental levels of toxic intermediates, which have been impli-cated in liver injury in patients with genetic bile salt synthesis defects [43]. Toxic effects of such intermediates may be pre-vented by FXR agonism, and may contribute to its favorable actions in drained animals.

Additional protection may be conferred by preservation of intestinal integrity, thus, limiting first pass exposure of the liver to dietary and microbial insults. Impaired gut barrier function following EBD is evident from elevation of circulat-ing LBP levels, which is already apparent after three days (da-ta not shown) . Thus, translocation of microbial products ap-pears an early event in hepatic injury following EBD. However, INT-747 did not reduce serum LPB levels. Likewise, the re-ported anti-inflammatory effects of FXR agonism were not apparent from our analysis of hepatic inflammatory genes. Thus, the molecular pathways underlying the hepatoprotective action of INT-747 in drained animals remain elusive.

The pathogenesis of liver disease in patients with intestinal failure has remained largely unknown, with studies mainly focusing on the effect of parenteral nutrition [44-46]. The pre-sent study focused on the mechanism of liver damage in a model recapitulating only the loss of bile, with preserved flow of pancreatic juices. An intact entero-hepatic cycle and FXR appear to be crucial in maintaining normal liver function. Ag-onistic activation of FXR reduced loss of bile and prevented liver damage in animals with an interrupted entero-hepatic cycle. Insights from the present study indicate that FXR ago-nism may be a possible approach to prevent the development of IFALD. Findings in this study could also be applied to other clinical diseases in which the entero-hepatic cycle is inter-rupted. For example, patients with ECF develop liver damage as a result of loss of bile fluid [5]. Reinfusion of fluid may be performed but has practical difficulties related to the anatomy of the fistula trajectory. In addition to possibly preventing liver damage, supplementation of INT-747 could also be considered for clinical application to control fistula output in ECF patients. Such an intervention may limit loss of fluid in general, and loss of electrolytes in particular. This may be especially rele-vant for patients with a fistula output above 1.5 L per day, who will require nutritional supplementation, usually through the parenteral route.
